# miRTar: an integrated system for identifying miRNA-target interactions in human

**DOI:** 10.1186/1471-2105-12-300

**Published:** 2011-07-26

**Authors:** Justin Bo-Kai Hsu, Chih-Min Chiu, Sheng-Da Hsu, Wei-Yun Huang, Chia-Hung Chien, Tzong-Yi Lee, Hsien-Da Huang

**Affiliations:** 1 Institute of Bioinformatics and Systems Biology, National Chiao Tung University, Hsin-Chu 300, Taiwan; 2 Department of Biological Science and Technology, National Chiao Tung University, Hsin-Chu 300, Taiwan; 3 Department of Computer Science and Engineering, Yuan Ze University, Chungli 320, Taiwan

## Abstract

**Background:**

MicroRNAs (miRNAs) are small non-coding RNA molecules that are ~22-nt-long sequences capable of suppressing protein synthesis. Previous research has suggested that miRNAs regulate 30% or more of the human protein-coding genes. The aim of this work is to consider various analyzing scenarios in the identification of miRNA-target interactions, as well as to provide an integrated system that will aid in facilitating investigation on the influence of miRNA targets by alternative splicing and the biological function of miRNAs in biological pathways.

**Results:**

This work presents an integrated system, miRTar, which adopts various analyzing scenarios to identify putative miRNA target sites of the gene transcripts and elucidates the biological functions of miRNAs toward their targets in biological pathways. The system has three major features. First, the prediction system is able to consider various analyzing scenarios (1 miRNA:1 gene, 1:N, N:1, N:M, all miRNAs:N genes, and N miRNAs: genes involved in a pathway) to easily identify the regulatory relationships between interesting miRNAs and their targets, in 3'UTR, 5'UTR and coding regions. Second, miRTar can analyze and highlight a group of miRNA-regulated genes that participate in particular KEGG pathways to elucidate the biological roles of miRNAs in biological pathways. Third, miRTar can provide further information for elucidating the miRNA regulation, i.e., miRNA-target interactions, affected by alternative splicing.

**Conclusions:**

In this work, we developed an integrated resource, miRTar, to enable biologists to easily identify the biological functions and regulatory relationships between a group of known/putative miRNAs and protein coding genes. miRTar is now available at http://miRTar.mbc.nctu.edu.tw/.

## Background

MicroRNAs (miRNAs) are small non-coding RNA molecules that are ~22 nts sequences capable of suppressing protein synthesis. Deriving from ~70-120 nts precursor transcripts that fold into stem-loop structures and thought to be highly conserved in genome evolution, miRNAs regulate 30% or more of the human protein-coding genes [1,2]. Moreover, previous investigations suggest that miRNA target sites in mammalians are preferentially conserved in mRNA sequences, especially in 3' UTR [3]. Since these miRNA-regulated genes are involved in various crucial cell processes including apoptosis, differentiation and development, Gene Ontology (GO) or Kyoto Encyclopedia of Genes and Genomes (KEGG) pathways enrichment analysis are helpful in understanding the biological functions of miRNA [4-7]. For instance, the target genes of miR-124a such as ephrins B1, B2, and B3, ephrin receptors A2, A3, and B4, semaphorins 5A, 6A, 6C, and 6D, and plexins A3 and B2 are involved in nervous system development in the axon guidance pathway.

Our previous work, miRTarBase [8], which is the most updated collection of miRNA-target interactions (MTI), has accumulated 3,576 experimentally verified MTIs between 657 miRNAs and 2,297 target genes among 17 species by means of manually surveying pertinent literature. Moreover, numerous computational programs are available for identifying miRNA target sites. TargetScan [9], miRanda [10] and RNAhybrid [11] are three computational tools for determining the most energetically favored hybridization sites of small to large RNAs. PicTar [12] is capable of identifying common targets of known miRNAs. DIANA-microT [13] system utilizes experimentally derived miRNA/mRNA binding rules. miRNAMap [14,15] and miRecords [16], miRGen [17,18] and GOmir [19] provide the putative miRNA-target interactions by combining prediction from multiple programs.

The miRU [20], MicroInspector [21], RNA22 [22], EIMMO [6], StarMir [23] and MMIA [24] are web-based tools for identifying miRNA binding sites. MicroInspector can search miRNA binding sites for a user-defined target RNA sequence that is potentially regulated by an miRNA. MicroInspector allows for variations in temperature and energies and allows the selection of various miRNA databases to identify miRNA binding sites of different strengths. The miRU tool was developed to predict plant miRNA target genes in any plant that is likely to be regulated by a user-defined miRNA. The pattern-based approach incorporated in the RNA22 program identifies putative target sites independent of miRNA target conservation and calls these sites as 'target islands'. The EIMMO considers evolutionary distance and branching when scoring the degree of miRNA target conservation. Furthermore, Dang *et al*. posited the target structure-accessible model for predicting miRNA targets and could also be accessed on system called StarMir. MMIA combines the inverse expression profiles of miRNA and mRNA data and then predicts the target genes by TargetScan, PicTar and PITA. Not only the aforementioned targeting of the 3' UTR of transcripts, but also the possibility of the targeting by miRNA of the coding sequence (CDS) and 5'UTR regions of the transcripts, are the subject of extensive research [2,22,25-35]. Indeed, more than twenty miRNA target prediction tools were developed to identify potential candidates for miRNA-target interactions. However, most of them do not provide convenient functions for biologists in exploring the biological functions and regulatory relationships between miRNAs and protein coding genes. The comparison between the different miRNA target prediction tools are given in Table 1.

**Table 1:** The comparisons of miRNA target prediction tools.

Features	miRTar	**DIANA-microT/miRPath **[47,68]	**EIMMO **[6]	**miRU **[20]	**RNAhybrid **[11]	**STarMir **[23]	**RNA22 **[22]	**MMIA **[24]
**Species**	Human	Human and mouse	Vertebrates, nematode, fly	Plants	Human, nematodes, flies	-	Vertebrates, nematode, fly	Human

* **1 to 1**	+	+	+	-	+	+	+	-
								
* **1 to N**	+	1 to All genes	+	-	+	+	-	+
								
* **N to 1**	+	-	+	-	+	+	-	-
								
* **N to M**	+	-	+	-	+	+	-	+
								
* **All to M**	+	All miRNAs to 1	+	-	-	-	-	-
								
* **1 to KEGG**	+	+	-	-	-	-	-	-

**miRNA targets on alternatively splicing exon**	+	-	-	-	-	-	-	-

**miRNA targets from mRNA**	3'UTR, CDS, and 5'UTR	3'UTR	3'UTR	3'UTR, CDS, and 5'UTR	3'UTR	3'UTR, CDS, and 5'UTR	3'UTR, CDS, and 5'UTR	3'UTR

**Known miRNAs**	miRBase V15	-	miRBase V12	-	-	-	-	-

**Accessibility of target site**	+	-	-	-	-	Sfold	-	-

**Conservation of target site**	+	+	+	+	-	-	-	-

**Expression profile of miRNA**	-	-	-	-	-	-	-	+

**Expression profile of target**	-	-	+	-	-	-	-	+

* 1 to 1 means the relation of one miRNA and one gene; 1 to N means the relation of one miRNA to multiple interesting genes; N to 1 means the relation of N miRNAs and one gene; N to M means the relation of N miRNAs and M genes; All to M means the relation of all miRNAs and M genes; 1 to KEGG means the relation of one miRNA and the genes of the selected KEGG map.

RNA alternative splicing plays important roles to regulate the gene expression in many biological processes among eukaryotic species. Recent studies have shown that more than 50% of genes undergo alternative splicing in humans [36-38]. Additionally, some researchers have observed that appropriate splice variants are involved in several cellular and developmental processes, including gender determination, apoptosis, axon guidance, cell excitation and contraction [39]. Relatedly, inappropriate alternative splicing causes the genetic disorders, because the expression of disease-related genes, many of which encode influential proteins in cancer biology, including those that govern cell cycle control, proliferation, differentiation, signal transduction pathways, cell death, angiogenesis, invasion, motility and metastasis, become abnormal [39-42]. Moreover, generated spatio-temporal splicing variants can be divided into five classical forms, which are cassette exons, alternative 5' splice sites, alternative 3' splice sites, mutually exclusive exons and retained introns [39,43]. Furthermore, the variety of combinations of *cis*-elements and *trans*-factors make understanding this mechanism difficult [39,42,43].

In this work, we aim to provide an integrated resource to allow biologists to elucidate miRNA-target interactions affected by the alternative splicing considering that the location of miRNA target sites may be found in exons, which are alternatively spliced. Several previous investigations have studied the miRNA-target interactions affected by alternative splicing [25,26,31,32,34]. For instance, Duursma *et al*. reported that human DNA methyltransferase 3b (DNMT3b) gene can be repressed by miR-148 family [31] and that the miR-148 target sites are located in the DNMT3b exons, which is alternatively spliced. Furthermore, the gene set enrichment analysis (GSEA) for a group of genes, which are targeted by one or more miRNAs, can provide an effective viewpoint to elucidate the miRNA functions in different biological process and pathways [44,45]. Previous investigations also analyzed the functions of miRNAs, mapping their putative target genes in several specific pathways [46-48], potentially elucidating the regulation of these biological pathways by means of miRNAs.

This work introduces an integrated resource that provides multiple analyzing functions for miRNA target identification and for the study of miRNA-target interactions, including the regulatory relationship between one miRNA and one gene, one miRNA and multiple genes, multiple miRNAs and one gene, and multiple miRNAs and multiple genes. Besides, miRTar identifies miRNA target sites against 3'UTR, as well as the coding regions and 5'UTR. This resource provides the information concerning that miRNA-target interactions are regulated by alternative splicing. Additionally, miRTar performs a gene set enrichment analysis for miRNA-regulated gene sets to decipher possible roles of miRNA in biological process and pathways.

## Implementation

**Figure 1:**
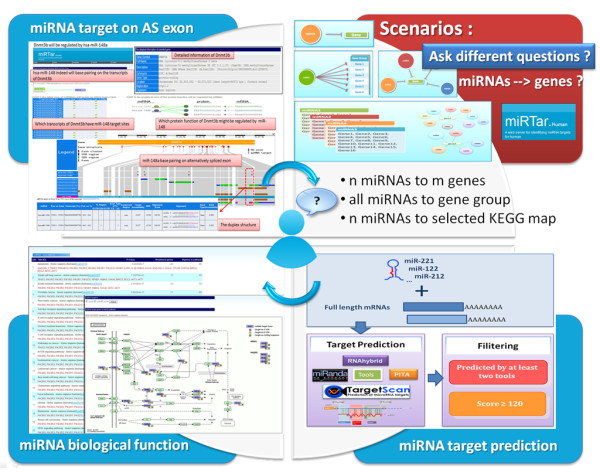
**Concept that underlies miRTar**. miRTar can provide several analyzing scenarios using which questions about the regulation by miRNAs of gene expression can be answered. It employs miRNA prediction tools to increase the probability of finding target sites. miRTar analyzes miRNA biological function using the KEGG pathway. It provides new information about the genetic regulation of miRNA by mRNA alternative splicing.

The miRTar is a web-based system that runs on an Apache web server with a Linux operating system. Figure 1 presents in brief the intention that underlies miRTar, which is to design an analytical platform that allows researchers to focus on all possible scenarios in order to discuss the regulatory relationships between miRNAs and genes. After data are submitted to the system, miRTar identifies the miRNA target sites using TargetScan, miRanda, PITA, and RNAHybrid. The miRTar identifies the target sites against 3' UTR, 5' UTR and coding regions. Thus, the potential miRNA-target interactions between miRNAs and genes are constructed. For a gene set that may be regulated by a single miRNA, based on the gene set enrichment analysis (GSEA), a p-value is calculated to estimate the overrepresentation of genes in the KEGG pathways, to estimate the biological function of miRNA. Additionally, miRTar is able to provide the information of miRNA target sites within exons, which could either be alternatively spliced (AS) or constitutively spliced (CS).

### Data collection

**Figure 2:**
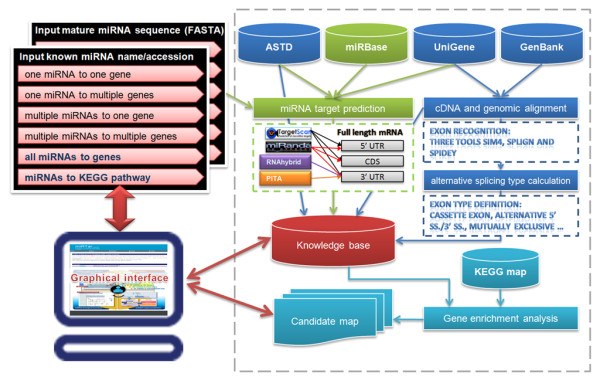
**System flow of miRTar**. miRTar system flow is composed of six parts. First, the various analyzing scenarios are designed. Second, data are collected from external database. Third, miRNA target sites are predicted. Fourth, exon/intron boundaries and alternatively spliced exon types are recognized. Fifth, the enrichment of the gene group is analyzed. Finally, a user-friendly graphical representation is designed to capture the regulation by miRNAs of genes.

Figure 2 depicts the system flow of miRTar. miRTar utilizes several well-known resources, including the miRNA sequences, obtained from miRBase database Release 15 [49], gene information and relevant annotations, based on ASTD database Release 1.1 [50] and GenBank database Release 167 [51]. The splice variants of transcripts are obtained from this ASTD [50], UniGene database Release 217 [52] and GenBank database [51]. The biological pathways are extracted from the KEGG/PATHWAY database Release 53.0 [53]. Table 2 lists all versions and data types obtained from external data sources, and the statistics concerning the data in the proposed resource.

**Table 2:** Data statistics and data obtained from databases.

Data source	Version	Data descriptions	Data amount
**miRBase **[49]	V.15	MicroRNA information (name, sequences,...)	1100

**KEGG **[53]	V. 53	The pathway maps	195

**ASTD **[50]	V. 1.1	Gene annotation	16,715
		mRNA sequences	93,467
		Protein information	34,545
		Alternative splicing events	78,165

**GenBank **[51]	V. 167	Gene annotation	32,123
		Genomic sequences	32,123
		Protein sequences	125,259

**UniGene **[52]	V. 217	mRNA sequences	137,654
		protein information (mRNA gi to protein gi)	125,259

### Identifying miRNA target sites in human

First, TargetScanS was utilized to detect perfect Watson-Crick base pairing against all mRNA transcripts with lengths of at least six nucleotides. Four seed types, 8mer, 7mer-m8, 7mer-A1 and 6mer, which were defined clearly by the Bartel's group [1]. Detecting the perfect seed region considerably reduces the number of false-positive predictions, especially for the conserved seed types [1,2,54]. The latest version of miRanda [55] is also utilized to identify miRNA target sites. Notably, the terminal miRNA nucleotides - the first and last two nucleotides, no longer contribute to the miRanda score [56]. The cutoff of minimal free energy (MFE) of the miRNA:target duplex was set to -12 kcal/mol and the cutoff of miRanda score was set to 120. Hence, miRNA targets having MFEs lower than -12 kcal/mol and a score that exceeds 120, are identified in the miRTar. On the other hand, RNAhybrid and PITA, which were developed to identify the miRNA target sites against 3'UTR, were utilized herein to identify miRNA target sites within 3'UTR. In order to reduce false positive predictions generated by multiple miRNA target prediction tools, miRTar applies several criteria concerning both their biological evolution and their structural context. These criteria are described below.

#### A. Target site in conserved region

Since target sites that are conserved across species are likely to be biologically functional, they are potential miRNA target sites. The UCSC PhastCons conservation score [57] is utilized to filter out the non-conserved predictions. Human data alignments were downloaded from the UCSC Genome Browser [58]. The lowest bound on the PhastCons conservation score at the predicted target site in a human is set to 0.5.

#### B. Target site in accessible regions

Conventional target prediction tools consider the complementarity between the miRNA and its target sequence, the conservation of the target sites, and the kinetics and thermodynamics of the miRNA/target duplex. Although these properties are important in identifying miRNA target sites, the sequence context surrounding miRNA target sites reportedly affects the binding affinities and the regulation of miRNAs. Harlan *et al .*[59] hypothesized that single-strand miRNAs can only bind to stretches of free mRNA for potential target sites. Dang *et al .*[23] posited the target structure-accessible model for predicting miRNA targets and succeeded in interpreting published data concerning the *in vivo *activity of C. *elegans *reporter genes that contain modified lin-41 3'-UTR sequences. In this work, the RNAplfold [60] program was employed to manifest the concept of target site accessibility to reduce the number of false positive predictions. Therefore miRNAs hybridize to the target sites, which are more likely to be real if they are in more accessible regions. RNAplfold can exactly determine the local base-pairing probabilities and the accessibilities of mRNA transcripts, which thus do not have to be computed from a Boltzmann-weighted sample of structures.

### Exon/Intron boundary recognition

Recognition of the boundaries between exons and introns in gene transcripts has been studied for several years. Numerous technologies have been adopted to align cDNAs against genomic sequences. In this work, the cDNA sequences are obtained from UniGene and the genomic sequences are obtained from GenBank [51]. Three tools are utilized to recognize these boundaries: SIM4 [61], splign [62], and spidey [63]. The exon/intron boundaries on the transcripts were confirmed by using at least two tools. A total of around one million exons from 150,000 transcripts in about 30,000 genes were recognized.

### Identifying different types of alternatively spliced exons

Five well-defined types of alternatively spliced exons are skipped exons, alternative 5' spliced sites, alternative 3' spliced sites, mutually exclusive exons and retained introns [43]. In this work, in order to identify different exon types, the collected transcripts from UniGene were aligned in a pairwise manner. First, the mRNA sequence was converted into a bit string of ones and zeros. Next, the logical operation (XOR, AND, OR), as discussed in SpliceInfo, is performed [64]. Furthermore, alternatively spliced exons from ASTD [50] can be downloaded from the website. Among these five types of alternatively spliced exons, the cassette exon has the most occurrence, followed in order by the alternative 5' splice sites and the alternative 3' splice sites; retained introns have the least (Table 3).

**Table 3:** Statistics of the various types of alternative splicing exons between two different data sources.

	Data source	
	
	ASTD	UniGene
**No. of cassette exon**	34,435	9,361,222

**No. of alternative 5' splice sites**	6,469	1,030,325

**No. of alternative 3' splice sites**	3,720	913,112

**No. of mutually exclusive exon**	3,384	9,401

**No. of intron retention**	9,639	75,481

### Effects of Alternative splicing to miRNA regulation

Following the prediction of miRNA target sites against all human transcripts, alternative splicing information were considered for elucidating the miRNA-target interactions affected by alternative splicing. We utilize two data sets of alternatively spliced exons to study how alternative splicing mechanism controls miRNA-target interactions. The first data set was obtained from ASTD [50] and the second data set was derived from the gene annotation in UniGene [52] and GenBank [51].

Table 4 presents the percentage of putative miRNA targets that are located on the transcripts that have been collected by miRTar. Since the average length of CDS is larger than the average length of 5' UTR and 3' UTR, generally the miRNA target sites are more probable to occur within the CDS regions than within 5' UTR and 3' UTR. Moreover, Table 5 gives the distributions of miRNA target sites within different types of alternatively spliced exons. The miRNA target sites are identified more often in cassette exons higher than in other types of alternatively spliced exons. The distribution is similar to the percentages of splicing exons given in Table 3. Accordingly, miRNA target sites located in alternatively spliced exons of a specific gene presents various potential regulatory relationships between the miRNA and the gene which can be further investigated. Thus, if an miRNA targets an alternatively spliced exon, the target site can be conditionally spliced out and cannot be included in the gene transcripts. Therefore, RNA alternative splicing can cause incomplete gene suppression by an miRNA and affect miRNA regulations in diverse protein functions.

**Table 4:** Statistics of miRNA target site locations

	Transcripts from ASTD	Transcripts from UniGene
*MFE	< = -12 kcal/mol	

*Score	> 120	

**5'UTR**	10.46%	9.32%

**CDS**	67.12%	65.83%

**3'UTR**	22.41%	24.85%

* miRNA target prediction parameters MFE: Minimum Free Energy of duplex; Score: alignment score of duplex.

**Table 5:** Statistics of miRNA target sites within different types of alternatively spliced exons between two different data sources.

		Data source	
		
		ASTD	UniGene
*MFE		< = -12 kcal/mol	

*Score		> 120	

**AS	**cassette exon**	11.98%	52.70%
			
	**alternative 5' SS**.	0.76%	6.80%
			
	**alternative 3' SS**	0.99%	7.55%
			
	**mutually exclusive**	0.92%	0.19%
			
	**intron retention**	3.92%	1.26%

**CS (Constitutively spliced exon)**		81.43%	31.50%

*miRNA target prediction parameters: MFE (Minimum Free Energy of duplex); Score (alignment score of duplex).

**AS: alternatively spliced exon.

### GSEA for miRNA-regulated genes

After the prediction of miRNA targets, miRTar performs a gene set enrichment analysis (GSEA) for the miRNA-regulated genes in the KEGG pathway maps. It allows users to conveniently observe the biological pathway in which the miRNA-regulated genes participate, and to determine the regulatory networks of miRNA-regulated genes.

**Figure 3:**
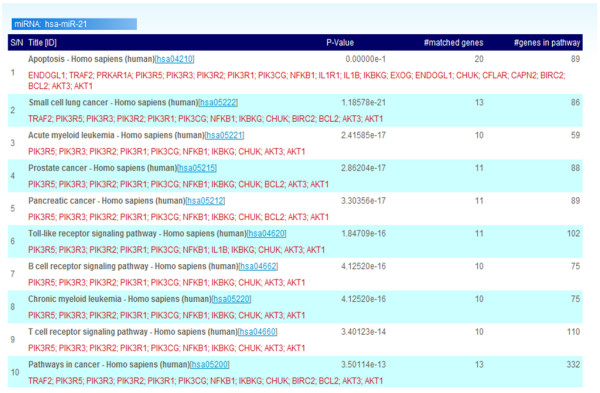
**Analysis to identify miRNA target genes in KEGG pathway maps**. Maps are ranked by the number of p-values of miRNA target genes in the pathway. Title (Map ID): miRNA targeted genes. Matched genes: Number of miRNA target genes. Genes in pathway: All genes.

As shown in Figure 3, the first step of the analysis is to determine the enrichment of specific miRNA target gene groups in various KEGG pathway maps. These maps are ranked by the number of p-values of the miRNA target genes in the biological pathway. The "Title [ID]" column provides the names of the KEGG pathway maps in which the miRNA target genes are involved; the "matched genes" column presents the number of miRNA target genes in each map; and the "gene in pathway" column presents all of the genes in each map.

**Figure 4:**
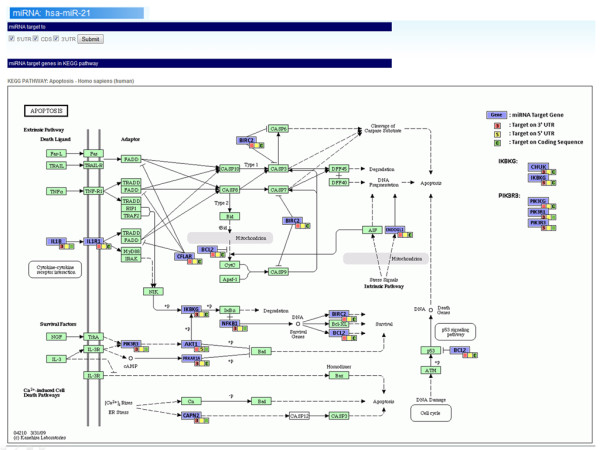
**miRNA target genes in KEGG pathway map**. Colors represent miRNA target genes and status of target region in KEGG pathway map. Red square: miRNA target on 3' UTR. Yellow square: miRNA target on 5' UTR. Green square: miRNA target on coding sequence. Purple square: miRNA target gene.

Figure 4 shows the second step of the analysis. The miRNA target genes are marked in "slate blue" in the KEGG pathway map, and the colors of traffic lights are utilized to represent the states of the miRNA target regions (3' UTR, 5' UTR and CDS). Users can focus in observing the miRNA target region of interest through changing the state in a biological pathway.

### The approximate runtime of miRTar

Users can identify the miRNA targets on a set of groups of genes by using multiple miRNA sequences. The execution time of ten randomly selected miRNAs against the gene set (in FASTA format around 20 MB file sizes) was computed on a PC server with eight CPU-cores. The miRNA target genes were predicted on average in 8.38 s for each miRNA, indicating that the proposed method can be utilized to identify the miRNA targets throughout the genome.

## Results

### Case study of alternatively spliced target-containing exon

To demonstrate the functionality of miRTar in realizing the functional interactions between mature miRNAs and alternative pre-mRNA splicing, the miRNA (miR-148) and the protein coding gene DNA methyltransferase 3b (Dnmt3b) were considered as a case study. Duursma et al's work [31] has shown that miR-148 can suppress Dnmt3b gene expression, targeting its protein coding region. One of its splice variants Dnmt3b3 mRNA lacks the target sites of miR-148. Additionally, the relative abundance of these splice variants results from the interactions between miRNAs and mRNA isoforms.

**Figure 5:**
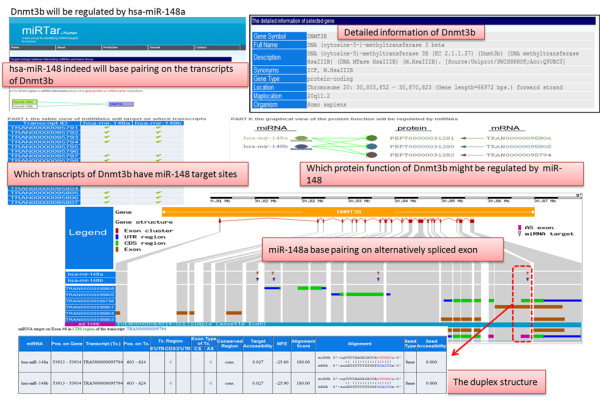
**miR-148 targets protein coding region of DNMT3b in human**. According to previous research [31], the miR-148 target is in the coding region of DNMT3b gene. miRTar yields results consistent with the experimental data.

Upon submission of the miRNA miR-148 and Dnmt3b gene using the miRTar web interface, miR-148 target sites prediction in all of the regions (5'UTR, CDS and 3'UTR) of gene transcripts is executed. Alternatively or constitutively spliced exons on the transcripts are annotated. Subsequently, based on the tables and graphs presented on the miRTar, miR-148 targets to CDS and 3'UTR of the Dnmt3b transcripts. The region of interaction is located in the alternatively spliced exons. Consequently, parts of the Dnmt3b transcripts can splice out the exon, resisting regulation by miR-148. The complementary sequences between of miR-148 and the transcripts are similar to those found in previous research [31]. Hence, miRTar has the potential power to elucidate the regulatory aspect of functional interactions (in which miRNA targets alternatively spliced exons), as shown in Figure 5.

### Case study of cancer-associated gene group

Analysis of the regulatory roles of miRNA in a biological pathway is one of the main functions of the miRTar system. Many studies have demonstrated that miRNA participates in various biological processes, including development, cell differentiation, proliferation, and apoptosis. In this work, the apoptosis-related properties of miRNAs and groups of genes are taken from research data [65] that have been established experimentally. To use the data as case study, KEGG pathway analysis of miRTar is carried out.

After the aforementioned data were submitted to miRTar, the results concerning the miRNAs indicated that each miRNA putatively regulates various gene groups by predicting the target on the transcripts of the genes. Additionally, the function of these gene groups in the biological pathway is associated with apoptosis. Hence, the results as shown in Figures 3 and 4 demonstrates that human miRNA hsa-miR-21 might be an important regulator in the apoptosis pathway when most of the biological functions of the target genes are involved in it. Many other miRNAs were also observed in this case study, but not shown in the Figures. The results can imply that the important regulatory roles of these miRNAs in the biological pathway are consistent with previous findings. Therefore, miRTar can be utilized to elucidate the possible function of miRNA in the KEGG pathways.

### Comparison with other miRNA target prediction web servers

The discovery of hundreds of miRNA genes has raised questions concerning how a specific miRNA regulates a specific gene, and what is the specific function of miRNA in a group of genes, among others. Most of miRNA target prediction tools can merely identify putative interactions between an miRNA and its targets, but they do not allow either gene set enrichment analysis for miRNA-regulated genes or the analysis of alternative splicing effects to miRNA-target interactions. Numerous analyzing scenarios, with various combinations of miRNAs and genes or KEGG maps input to miRTar can be considered to yield preliminary answers. Table 1 lists the comparisons among miRTar and other miRNA target prediction tools or web servers. The miRTar provides the most convenient way for miRNA target prediction analysis and presents the most plentiful information for miRNA-target interactions such as KEGG pathways and alternative splicing information. Besides, miRTar integrated several external databases in advance. For instance, the sequences and annotations of miRNAs and genes were stored in the resource. It only requires inputting the accessions for miRNA and genes into miRTar instead of inputting the sequences of miRNAs and genes, which should be prepared by the users when using other tools or web servers.

## Discussion

In this work, we aim to develop an integrated system for identifying miRNA-target interactions rather than to develop a new algorithm for identifying miRNA target genes. Further information including KEGG pathways and alternative splicing of genes were presented and analyzed. The miRTar system can identify putative miRNA target sites on transcript sequences of genes under the severe constraints that have been discussed in previous studies. In miRTar, the default parameters are set based on the analysis 972 known miRNA target sites, collected in miRTarBase [8]. Seventy-six percent of known miRNA-target interactions can be identified under the criteria MFE < = -7 and alignment score > = 125.

As given in Table 5, part of miRNA targets are located in alternative splicing exon regions that means the site in the exon of one mRNA isoform is recruited, but is not in another mRNA isofrom of the same gene. In this work, the proportion of this kind of target sites is larger than 50% in all of the predicted sites on human species. Therefore, when discussing the regulatory relationship between miRNAs and target genes, it is important to have another point of view in RNA alternative splicing. Accordingly, one of the directions is that the observation of miRNA base-pairing in the particular region of the gene-exon sequence may be comprised alternatively spliced exons. This information is useful in discussing the possible regulatory relationship between RNAi and RNA alternative splicing, which has been mentioned in previous investigations [31,66]. Notably, the prediction of miRNA targets in miRTar involves not only 3' UTR but also 5' UTR and CDS, implying that the protein products of one gene might also be repressed by the miRNA targeting of the CDS and UTR.

Another direction concerns the possible roles of miRNA in biological processes. Whereas several studies have identified genes that are regulated by miRNAs, the mechanisms of these miRNAs-associated mechanisms are not well known. Therefore, miRTar adopts the enrichment method in the KEGG pathway of the gene group to evaluate each the potential biological functions of miRNA. Moreover, when using miRTar to predict known miRNA target sites, some of them cannot be identified based on the default predictive parameters. For example, one research [67] shows that one of the experimental data can be targeted by hsa-let-7a on FOXA1, but miRTar can't detect any miRNA:target base pairing interaction in 3' UTR of gene transcripts (Additional file 1).

## Conclusions

The miRTar develops an integrated resource for deciphering miRNA-target interaction networks, and provides a broad range of analyzing scenarios for miRNA-target interactions, including one miRNA to one gene, one miRNA to multiple genes, and others, to help biologists understand the regulation between the miRNAs and target genes. By integrating several external databases and analyzing tools, miRTar can provide further information for elucidating miRNA regulation affected by alternative splicing. Besides, miRTar can enable biologists to easily identify the biological functions and regulatory relationships between a group of known/putative miRNAs and protein coding genes.

## Availability and requirements

The miRTar system is freely available at http://mirtar.mbc.nctu.edu.tw/human/.

## Authors' contributions

HDH conceived and supervised the study. JBKH, CMC, WYH and SDH were responsible for the design, computational analyses, implementation of the system, and drafting the manuscript. CHC and TYL were in charge of manuscript revision and data update. All authors read and approved the final manuscript.

## Supplementary Material

Additional file 1 **hsa-let-7a can target on FOXA1**. The previous research [67] shows the duplex structure of let-7a and the transcript of FOXA1. For the standard seed criteria, only one wobble base pair is allowed in it. The duplex structure in this figure, however, there are too many wobble base pairs (shows in yellow color) in the seed region (shows in green color) to be skipped in the results of miRTar. Click here for file
