# Supplementary material for: miRTar: an integrated system for identifying miRNA-target interactions in human
Source: BMC Bioinformatics. 2011 Jul 26;12:300. doi: 10.1186/1471-2105-12-300 (PMC3162936; doi:10.1186/1471-2105-12-300)
Supplement: Additional file 1 — hsa-let-7a can target on FOXA1. The previous research [67] shows the duplex structure of let-7a and the transcript of FOXA1. For the standard seed criteria, only one wobble base pair is allowed in it. The duplex structure in this figure, however, there are too many wobble base pairs (shows in yellow color) in the seed region (shows in green color) to be skipped in the results of miRTar. [file 1471-2105-12-300-S1.PDF]

Figure S1. hsa-let-7a can target on FOXA1 [1]

The previous research [1] shows the duplex structure of let-7a and the transcript of FOXA1. For the standard seed criteria, only one wobble base pair is allowed in it. The duplex structure in this figure, however, there are too many wobble base pairs (shows in yellow color) in the seed region (shows in green color) to be skipped in the results of miRTar.

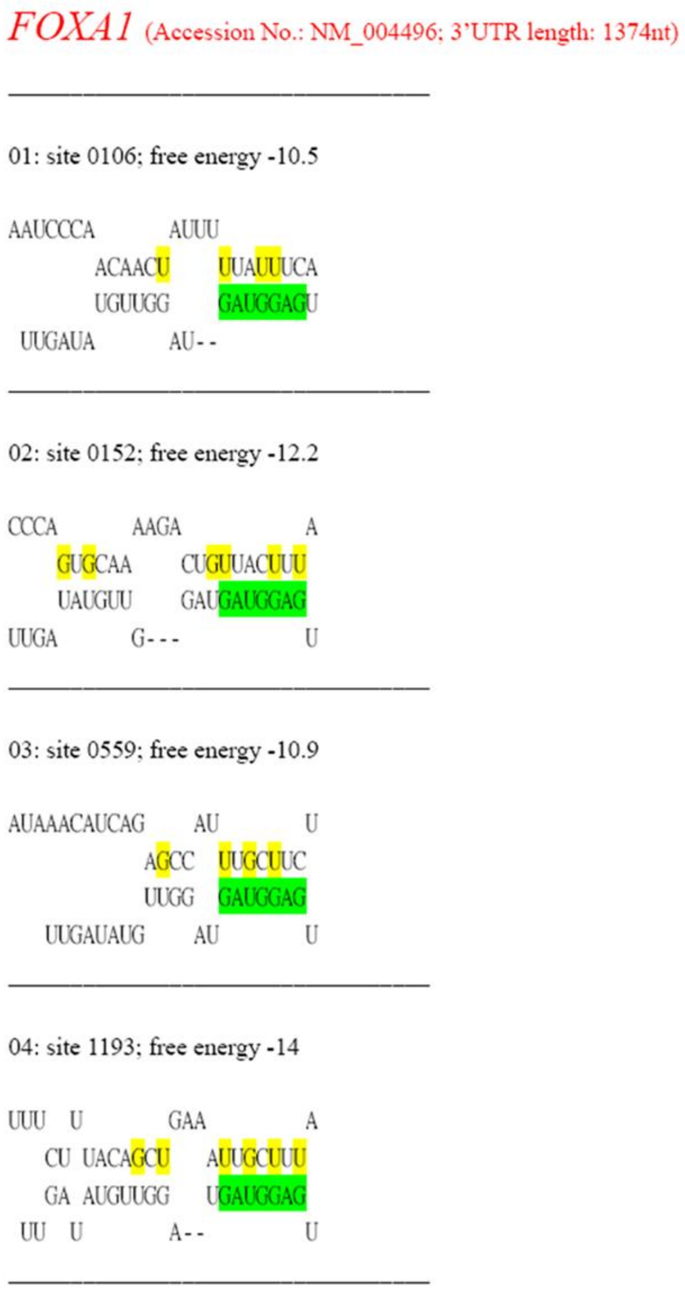

Figure S1.

1. Lin YC, Hsieh LC, Kuo MW, Yu J, Kuo HH, Lo WL, Lin RJ, Yu AL, Li WH: Human TRIM71 and its nematode homologue are targets of let-7 microRNA and its zebrafish orthologue is essential for development. *Mol Biol Evol* 2007, 24(11):2525-2534.
